# Enhancing RT‐PCR Throughput and Sensitivity through Large‐Scale Sample Pooling Using a Nano‐Hybrid Membrane

**DOI:** 10.1002/advs.202408771

**Published:** 2025-01-20

**Authors:** Na Eun Lee, Kang Hyeon Kim, Ji Hye Hong, Seungmin Lee, Jeong Soo Park, Dohwan Lee, Dae Sung Yoon, Jeong Hoon Lee

**Affiliations:** ^1^ Department of Electrical Engineering Kwangwoon University Seoul 01897 Republic of Korea; ^2^ Department of Biotechnology College of Life Sciences and Biotechnology Korea University Anam‐dong, Seongbuk‐gu Seoul 02841 Republic of Korea; ^3^ School of Biomedical Engineering Korea University Seoul 02841 Republic of Korea; ^4^ School of Mechanical Engineering Korea University 145 Anam‐ro, Seoungbuk‐gu Seoul 02841 Republic of Korea; ^5^ Interdisciplinary Program in Precision Public Health Korea University Seoul 02841 Republic of Korea; ^6^ Astrion Inc. Seoul 02841 Republic of Korea; ^7^ CALTH Inc. Changeop‐ro 54, Seongnam Gyeonggi 13449 Republic of Korea

**Keywords:** group testing, high‐throughput RT‐PCR, sample enrichment, sample pooling, SARS‐CoV‐2

## Abstract

During the pandemic surge, including SARS‐CoV‐2 and influenza, pooling samples emerged as an efficient strategy to identify infected individuals in large groups. While pooling enhances RT‐PCR throughput, reducing costs and resources, it dilutes positive samples with negative ones, lowering sensitivity and increasing false negatives. This study proposes a new method to address the trade‐off between pool sizes and RT‐PCR accuracy. This method integrates large‐scale pooling with sample enrichment using a nano‐hybrid membrane, preventing the pooling‐induced decrease in viral concentration and maintaining cycle threshold (Ct) values close to individual positive samples. The nano‐hybrid membrane, named SIMPLE (streamlined, simple, and inexpensive method for preconcentration, lysis, and nucleic acid extraction), comprises layered red blood cell membranes, polyethersulfone, and silica membranes. Using SIMPLE, a Ct value reduction to ≈2.6 is demonstrated in pooled COVID‐19 samples with a pool size of 6 and found Ct values from larger pool sizes (8, 16, 32, 64, and 128) comparable to individual positive samples.

## Graphical abstract

This study enhances RT‐PCR sensitivity and throughput by employing large‐scale sample pooling with a nano‐hybrid membrane. This membrane features a layered structure of red blood cell membrane‐coated polyethersulfone combined with silica membranes, effectively concentrating viral samples and mitigating dilution effects in pooled samples. This innovation supports efficient outbreak management and rapid diagnostics, contributing to improved public health responses.

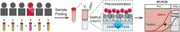

## 1 Introduction

The COVID‐19 pandemic, caused by severe acute respiratory syndrome coronavirus 2 (SARS‐CoV‐2), unfolded as a global catastrophe, placing unprecedented burdens on global healthcare systems and unleashing profound public health risks and economic crises.^[^ ^1^ ^]^ At the peak of the pandemic in January 2021, the United States (US) reported its highest weekly incidence of hospitalization and deaths, with over 1 30 000 new hospital admissions and ≈25 000 deaths per week (data obtained from COVID Data Tracker, the Centers for Disease Control and Prevention). The global situation mirrored this dire scenario, with worldwide weekly hospitalization and death rates peaking at ≈200 000 cases and ≈51 000 deaths during the same period (data obtained from COVID‐19 Data Explorer, Our World in Data).

As virus transmission was unexpectedly rapid and widespread, the economic impact was equally staggering. The average cost of hospital care per patient in the US was estimated at $78, 569, with more than 30% of the total cost attributed to diagnostic testing.^[^ ^2^ ^]^ If we assume each individual is tested by reverse transcription polymerase chain reaction (RT‐PCR) with no grouping, the estimated total daily costs in January 2021 in the US could exceed $28 million; this estimate is based on 1) ≈$17 for RT‐PCR testing per individual,^[^ ^3^ ^]^ 2) ≈200 000 daily confirmed COVID‐19 cases in the US, and 3) ≈12% positive rate during the same period (data collected from COVID‐19 Data Explorer). Furthermore, the pandemic surge severely strained other resources, including medical workers and supply chains for personal protective equipment, testing reagents, sample swabs, and laboratory equipment.^[^ ^4^, ^5^ ^]^

Given these significant challenges, the necessity for high‐throughput RT‐PCR testing became evident, leading to the adoption of sample pooling or group testing techniques.^[^ ^6^, ^7^ ^]^ Sample pooling, a method that enhances the throughput of testing a large number of samples using fewer resources, can significantly contribute to the timely identification and isolation of infected individuals, preventing the spread of infectious diseases. It involves grouping (i.e., mixing) individually collected samples—such as nasopharyngeal or oropharyngeal swabs in viral transport medium (VTM) or saliva—and testing them with a single RT‐PCR, that has been widely adopted following the Food and Drug Administration's first Emergency Use Authorization for sample pooling in COVID‐19 testing in July 2020. High‐throughput RT‐PCR through pooled samples can significantly contribute to the timely identification and isolation of infected individuals, thereby preventing the international spread of infectious diseases. The simplest scheme, Dorfman pooling, accommodates 4–32 samples per test. The substantial reduction in cost and resource use through sample pooling has been reported to range from ≈47% to 80%, depending on the pooling size and the prevalence rates.^[^ ^3^, ^8^, ^9^ ^]^

While sample pooling strategies are highly effective, they typically raise significant concerns regarding sensitivity and the risk of false negatives due to sample dilution.^[^ ^9^, ^10^, ^11^ ^]^ When samples are pooled, the viral load in each individual sample is diluted, leading to an increased cycle threshold (Ct) value in RT‐PCR testing.^[^ ^12^, ^13^ ^]^ For example, pooling a single positive sample with a low viral load (Ct value = 34) with seven negative samples (totaling eight in the pool) can theoretically increase the resultant Ct value to 37. Considering that Ct values greater than 35 are declared as negative in many SARS‐CoV‐2 RT‐PCR kits,^[^ ^14^ ^]^ this scenario can lead to failures in identifying positive cases, potentially missing infected individuals who carry low viral loads. This highlights the crucial trade‐off between increasing pool sizes for higher throughput and preserving the accuracy of RT‐PCR testing, a major issue that limits the indefinite increase in pooled sample sizes. Although many studies have suggested optimized mathematical schemes for sample pooling, the fundamental problem of increased Ct values due to dilution remains unresolved.^[^ ^15^, ^16^, ^17^ ^]^

To overcome this fundamental challenge and address this trade‐off, we introduce an innovative method that simultaneously enriches and extracts nucleic acids from pooled samples using a nano‐hybrid membrane. The nano‐hybrid membrane, a combination of layered red blood cell membranes (RBCM), polyethersulfone (PES), and silica membranes, is referred to as SIMPLE, which stands for the streamlined, simple, and inexpensive method for preconcentration, lysis, and nucleic acid extraction. Without loss of generality, we built the SIMPLE on conventional spin columns primarily used in molecular biology, SIMPLE enables seamless adoption in standard laboratories without additional procedures or cost. The spin column equipped with SIMPLE enriches the pooled samples and subsequently extracts and purifies the enriched nucleic acids. This enrichment process prevents the dilution‐induced decrease in viral load concentration, ensuring the absolute amount of viral load within the pooled sample is available for RT‐PCR testing. This approach maintains the Ct values of pooled samples closely approximated with those of individual positive samples, potentially breaking the limitations of increasing the size of sample pooling, thus enhancing the throughput of RT‐PCR regardless of the pooling size. Furthermore, the subsequent extraction and purification on SIMPLE enables the efficient elution of the enriched nucleic acids, preserving the sensitivity and accuracy of RT‐PCR testing. Using this method, we demonstrated 1) a Ct value reduction to ≈2.6 in the pooled COVID‐19 patient samples with a pool size of 6 (one positive and five negative samples), and 2) results with considerably larger pool sizes of 8, 16, 32, and 64, showing the Ct value reduction to the level comparable to that obtained from individual positive samples (all with one positive and the rest negative samples).

## 2 Result and Discussion

### 2.1 Operation of the SIMPLE to Process Pooled Sample

**Figure 1:**
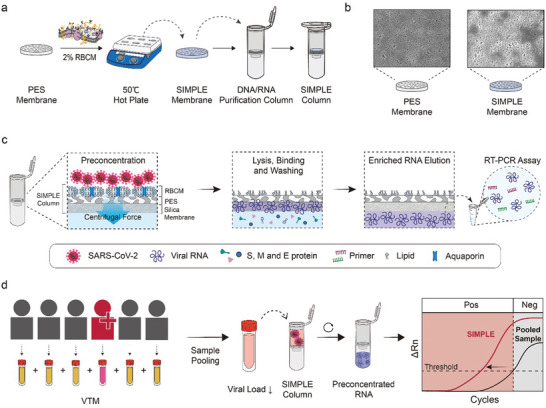
Operation of the SIMPLE in Processing Pooled Samples. a) The SIMPLE, comprising stacked layers of RBCM, PES, and silica membranes, integrated into a conventional spin column. b) Scanning electron microscope images of the PES and RBCM‐coated PES membranes. c) Under centrifugal force, liquid from the pooled sample is rapidly expelled, while virions are enriched at the top of the SIMPLE. The enriched/purified viral RNAs are subsequently eluted from the SIMPLE for RT‐PCR testing. d) Utilizing the SIMPLE column mitigates the dilution‐induced decrease in viral concentration, a primary concern in sample pooling strategies. The Ct value of the sample processed by the SIMPLE closely aligns with that of the original positive sample(s), enhancing RT‐PCR throughput without sacrificing sensitivity.

The SIMPLE comprises stacked layers of RBCM, PES, and silica membranes (see details in the Materials and Methods) and is integrated into a conventional spin column to maintain the generality of biological experiments (**Figure** **1**a). This integration facilitates seamless adoption in any standard laboratory. The upper two layers, consisting of RBCM‐coated PES (scanning electron microscope images, Figure 1b; Figure S1, Supporting Information), are responsible for sample enrichment, while the bottom silica membrane handles nucleic acid extraction. The PES membrane was specifically chosen for its adaptable properties: it offers a range of pore sizes, exceptional mechanical strength and flexibility for easy shaping, and high resistance to pressure‐induced fractures. Unlike standard size‐based filters, the RBCM coating membrane provides tunable permselectivity due to the presence of aquaporin water‐transporting proteins,^[^ ^18^ ^]^ which enable rapid water transport and enhance sample enrichment efficiency. X‐ray photoelectron spectroscopy (XPS) analysis confirms surface elemental changes following RBCM coating on the PES membrane. In the bare PES membrane, primary surface elements included carbon, oxygen, and sulfur, reflecting its polyether and sulfone chemical structure. In contrast, the RBCM‐coated PES membrane exhibited additional phosphorus and nitrogen peaks, indicating the presence of biomolecular elements (Figure S2a, Supporting Information).

By applying centrifugal force to the pooled sample loaded into the SIMPLE‐equipped spin column (SIMPLE column), the liquid sample is rapidly withdrawn due to the high flux of the PES layer and the aquaporin water‐transporting proteins on the RBCM (Figure 1c), which provide a major pathway for rapid water transportation.^[^ ^18^ ^]^ Meanwhile, virions in the pooled sample are preconcentrated at the top of the SIMPLE, ensuring that all virions contained in the sample are available for subsequent assay, even when the samples are severely diluted due to large pool sizes. After enrichment, lysis and binding buffers are added to the SIMPLE column to lyse the enriched virions and extract the RNAs through the silica membrane at the bottom. During this process, the RBCM coated on the PES is also lysed (Figure S1¸ Supporting Information). The released viral RNAs exhibit minimal non‐specific binding to the PES membrane, with only ≈5% of the RNA binding non‐specifically, and the majority of RNA is captured by the silica membrane (Figure S2b, Supporting Information). The captured RNAs on the silica membrane are subsequently washed, and the enriched/purified RNAs are eluted with deionized water (D.W) for subsequent RT‐PCR testing. All buffers used are discarded by applying centrifugation to the SIMPLE column.

This approach using the SIMPLE column effectively prevents the dilution‐induced decrease in viral concentration that occurs when a single or a few positive samples are pooled with multiple negative samples (Figure 1d). It enables maintaining the Ct values of pooled samples close to those of the original positive sample(s), potentially improving the throughput of RT‐PCR without compromising its sensitivity and accuracy, i.e., the rate of false negative results. For instance, when a single positive sample with a low viral load (Ct value = 34) is pooled with five negative samples (pool size = 6), the Ct values would theoretically increase to 36.6 when using a conventional column, which is beyond the typical positive declaration range (Ct value < 35). This would lead to the assay mistakenly identifying the pooled sample as negative, resulting in a false negative result. However, the SIMPLE can enrich the pooled sample, mitigate the dilution effect from pooling, and thereby reduce the resultant Ct value to a level comparable to that of the unpooled positive sample (Ct value = 34).

### 2.2 Evaluation of Enrichment Performance in the SIMPLE

**Figure 2:**
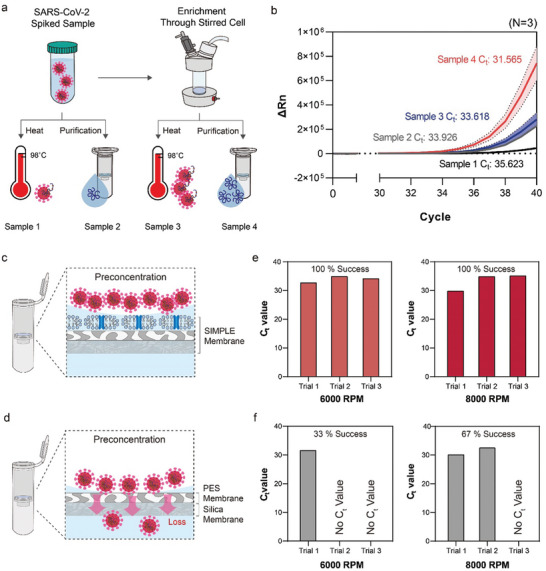
Evaluation of Enrichment Performance Using the SIMPLE. a) Preparation of samples spiked with inactivated SARS‐CoV‐2 particles for four different treatments: Sample 1: heat‐lysed sample, Sample 2: sample with viral RNA extracted, Sample 3: SARS‐CoV‐2 enriched, resuspended, and heat‐lysed, and Sample 4: SARS‐CoV‐2 enriched, resuspended, and extracted. b) Ct values obtained by RT‐PCR for the four samples, with error bars (red and blue spread) indicating the standard deviation (N = 3). c) Schematic of the SIMPLE column. d) Schematic of the column lacking the RBCM coating. e) Ct values from RT‐PCR used to evaluate the efficacy of combined enrichment and purification in the SIMPLE column. f) Ct values from the column lacking the RBCM coating, assessing the impact on performance.

The enrichment capability of the RBCM‐coated PES, which constitutes the upper two layers of the SIMPLE, was evaluated via RT‐PCR. This evaluation involved measuring the changes in Ct values with and without enrichment. For this purpose, 1 mL samples spiked with inactivated SARS‐CoV‐2 particles in 1X PBS were prepared and subjected to four different treatments (**Figure** **2**a): (1) samples were heat‐lysed at 98 °C for 10 min, (2) viral RNAs were extracted using a conventional spin column, (3) SARS‐CoV‐2 was enriched using the RBCM‐coated PES, resuspended in 100 µL, and subsequently heat‐lysed at 98 °C for 10 min, and (4) SARS‐CoV‐2 was enriched, resuspended in 100 µL, and subsequently extracted using the conventional spin column. The thermal lysis was introduced to verify viral enrichment prior to proceeding the purification process. The enrichment was performed by placing the RBCM‐coated PES in the stirred cell.

RT‐PCR was conducted on all treated samples (*N* = 3), and their Ct values were compared (Figure 2b). As anticipated, sample 4 exhibited the lowest Ct value (31.565) among the others, benefiting from both enrichment and RNA purification. In contrast, sample 1 exhibited the highest Ct value (35.623), higher than sample 2 (33.926), probably due to the presence of PCR inhibitors in the viral debris. Notably, sample 3, which was enriched but not purified, showed a slightly lower Ct value (33.618) than sample 2. These results suggest that the RBCM‐coated PES, the upper two layers of the SIMPLE, can effectively enrich the virus, and the effectiveness of this enrichment is comparable to that of RNA extraction/purification from an RT‐PCR perspective.

Next, we assembled the SIMPLE column by stacking the RBCM‐coated PES with a silica membrane and incorporating these layers into a spin column (Figure 2c). We subsequently evaluated the efficacy of the SIMPLE column for combined enrichment and purification on a single platform. As a control, we used a column identical in design but lacking the RBCM coating (Figure 2d). The primary objective of this experiment was to verify that both the enrichment layer (RBCM‐coated PES) and the RNA purification layer (silica membrane) function effectively when integrated into a single monolithic membrane.

To conduct this evaluation, we processed 1 mL of 1X PBS samples spiked with inactivated SARS‐CoV‐2 particles through both columns by centrifugation at 6 000 and 8 000 RPM, followed by RT‐PCR amplification of the eluted RNAs. The results indicated that all samples processed through the SIMPLE column yielded successful RT‐PCR results at both centrifugation speeds (Figure 2e). In contrast, the column without the RBCM coating occasionally failed to produce RT‐PCR results (Figure 2f), with success rates of 33% at 6 000 RPM and 67% at 8 000 RPM. This is attributed to the smaller and more uniform pore sizes of the RBCM compared with the PES membrane. Without the RBCM coating, virus particles may pass through the PES membrane and be lost during centrifugation, compromising sample enrichment. Although these results demonstrate that the SIMPLE column can achieve both virus enrichment and RNA extraction within a single platform, its performance was slightly inferior (Ct value = 29.922, 34.908, and 35.199, Figure 2e) compared with when enrichment and extraction were conducted separately (Ct value = 31.565, Figure 2b). This suboptimal performance was attributed to the lack of optimization in RNA extraction protocols, specifically 1) the centrifugation RPM and 2) the removal of residual liquid samples on the silica membrane before adding the lysis and binding buffer.

### 2.3 Optimization of RNA Extraction Protocol in the SIMPLE

**Figure 3:**
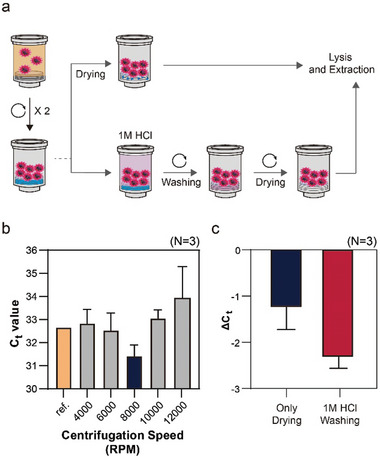
Optimization of RNA Extraction Protocol in the SIMPLE. a) Strategies for removing residual liquid from sample enrichment: 1) Drying by centrifugation and 2) Washing with 1 m HCl. b) Optimization of centrifugal RPM for drying the residual liquid sample, showing that the lowest Ct value (indicating the highest efficiency of RNA extraction) was achieved at 8 000 RPM. c) Impact of washing the silica membrane with 1 m HCl on residual liquid removal using 8 000 RPM. Error bars indicate the standard deviation (N = 3).

While the SIMPLE column demonstrated effective enrichment performance, the RNA extraction performance did not meet the results obtained when enrichment and extraction were performed separately. This shortfall was attributed to insufficient removal of residual liquid samples on the silica membrane after sample enrichment, which impeded the formation of cationic bridges necessary for RNA binding during the subsequent lysis and binding process (**Figure** **3**a). The residual liquid samples prevented the chaotropic salt from effectively charging the silica membrane. To address this, we implemented two approaches: 1) drying the residual liquid on the silica membrane before the RNA extraction steps using varying centrifugal RPMs, and 2) introducing a washing step with 1M HCl between the sample enrichment and RNA extraction steps (Figure 3a).

Initially, we attempted to dry the residual liquid on the silica membrane using different centrifugal RPMs ranging from 4 000 to 12 000 RPM prior to RNA extraction (Figure 3b). A 1 mL sample of 1X PBS spiked with inactivated SARS‐CoV‐2 particles was enriched under these conditions and extracted to obtain the enriched/purified RNAs. The eluted RNAs were subsequently amplified using RT‐PCR to evaluate the effect of RPM on RNA extraction efficiency. For comparison, an identical sample processed by the conventional spin column showed a Ct value of 32.657. All experiments were repeated three times (N = 3). Among the tested RPMs, 8 000 RPM yielded the lowest Ct value (31.414), indicating the highest RNA extraction efficiency. This was possible because the residual sample could not completely dry at lower RPMs (4 000 and 6 000 RPM), while at higher RPMs (10 000 and 12 000 RPM), virus particles were unintentionally lost by passing through the SIMPLE membrane.

Although 8 000 RPM was the most efficient among the RPM tests, the reduction in Ct value (1.243, Only Drying in Figure 3c) did not reach the expected value ( ≈2.3, reflecting a 5‐fold enrichment due to the five times greater sample volume in the SIMPLE compared to a conventional column). This suggested that the silica membrane's function was not fully restored. To enhance the silica membrane's RNA binding capability, we added a washing step with 500 µL of 1M HCl before introducing the lysis and binding buffer to thoroughly remove the residual sample and strengthen the formation of cationic salt bridges on the silica membrane.^[^ ^19^, ^20^ ^]^ As anticipated, the addition of 1M HCl significantly improved the binding efficiency of the silica membrane to RNAs, achieving a Ct value reduction to 2.31, which aligns with the ideal value (1M HCl Washing in Figure 3c). Taken together, these optimizations in the procedures required for RNA extraction in the SIMPLE effectively resolved the issue of weakened silica membrane binding due to residual samples, confirming the utility of the added washing step with 1 m HCl.

### 2.4 Assay on Pooled COVID‐19 Patient Samples Using the SIMPLE

**Figure 4:**
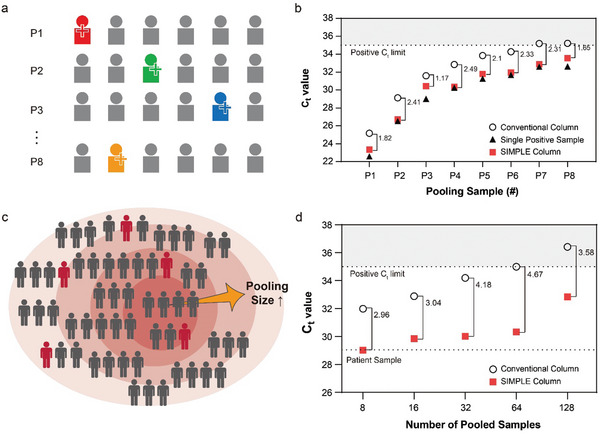
Assay on Pooled COVID‐19 Patient Samples Using the SIMPLE. a) Pooling methodology where each of eight single SARS‐CoV‐2 positive samples (Ct values ranging from 22.6 to 32.6) was pooled with five negative samples (pool size of 6). b) Comparison of Ct values from non‐pooled single positive samples, and pooled samples processed by both the conventional column and the SIMPLE column. c) Evaluation of massive sample pooling with pool sizes of 8, 16, 32, 64, and 128, each containing one positive sample (Ct value of 29) and the remaining negative samples. d) Ct values from samples of various pool sizes processed by the conventional column and the SIMPLE column are presented.

With the optimized protocol for sample enrichment and RNA extraction, we analyzed pooled COVID‐19 patient samples to evaluate the performance of the SIMPLE column. Nasopharyngeal swabs collected in VTM from patients served as samples (Table S1, Supporting Information). Initially, we identified SARS‐CoV‐2 positive samples through RT‐PCR, with Ct values ranging from 22.6 to 32.6. Thereafter, each PCR‐confirmed positive sample was pooled with five negative samples, creating a pool size of 6 (**Figure** **4**a). The pooled samples were processed using both the SIMPLE column and a conventional spin column (as a control), and their Ct values were measured through RT‐PCR.

As expected, the conventional spin column produced increased Ct values for the pooled samples, averaging ≈2.6 units higher than those of the original single positive samples (Conventional column, Figure 4b). In contrast, the SIMPLE column produced Ct values for the pooled samples that closely approximated those of the original single positive samples (SIMPLE column, Figure 4b), achieving an average Ct value decrease of 2.035 compared to the conventional column. Notably, samples P7 and P8, which were falsely identified as negative (Ct value > 35) by the conventional column, were correctly identified as positive by the SIMPLE column, thus preventing false negative results.

Following the analysis with 6‐pooled samples, we extended the evaluation to massive pooling with pool sizes of 8, 16, 32, 64, and 128 (Figure 4c). A single positive sample with a Ct value of 29 was pooled with 7, 15, 31, 63, and 127 negative samples, respectively, significantly diluting the viral concentration in the pooled samples from 8 to 128 times. After processing the massively pooled samples using both the SIMPLE column and the conventional spin column, we measured each Ct value using RT‐PCR. As anticipated, the Ct values for the pooled samples processed by the conventional column were significantly increased to 31.984, 32.882, 34.194, 34.998, and 36.415 for pool sizes of 8, 16, 32, 64, and 128, respectively (Conventional column, Figure 4d). Notably, samples pooled 64 times and 128 times were falsely identified as negative due to their Ct values exceeding 35. However, when processed by the SIMPLE column, their Ct values were reduced (SIMPLE column, Figure 4d), and even the 64‐pooled and 128‐pooled samples, which were identified as negative by the conventional column, were correctly identified as positive, significantly reducing the risk of false negatives. While the efficiency of Ct value reduction slightly decreased when processing the 128‐pooled sample (only 3.58), likely due to incomplete protein digestion caused by high protein content exceeding the capacity of added proteinase K, the other pooled samples demonstrated reductions in Ct values to levels comparable to the original single positive sample: reductions of 2.96, 3.04, 4.18, and 4.67 for pool sizes of 8, 16, 32, and 64, respectively. These findings indicate that the SIMPLE column, equipped with the optimized protocol, is capable of enriching the virus and maintaining the Ct values of massively pooled samples close to those of single positive samples. The results demonstrated that the risk of false negatives was significantly reduced, and the throughput of RT‐PCR testing was improved without compromising the accuracy of the RT‐PCR test.

## 3 Conclusion

This study introduces a new sample pooling method that effectively mitigates the trade‐off between pool size and RT‐PCR throughput using the SIMPLE column. This innovative method enhances the enrichment of viruses in pooled samples and facilitates the extraction of enriched and purified viral RNAs, thereby preventing the dilution‐induced decrease in viral RNA concentration typically associated with sample pooling. The application of the SIMPLE column ensures that Ct values of pooled samples remain close to those of individual positive samples, regardless of the pooling size. This capability was clearly demonstrated by the significant reduction of Ct values in pooled COVID‐19 patient samples, with pool sizes of up to 64, resulting in Ct values comparable to those of individual positive samples. This substantially reduces the risk of false negatives and enhances the throughput and accuracy of RT‐PCR testing. Unlike commercial purification kits, which are typically limited to processing volumes of ≈200 µL (Table S2, Supporting Information), SIMPLE can potentially handle large pooled samples, including pooling sizes of 128 or more, in a few spins when using a large‐capacity centrifuge, given its compatibility with various spin column sizes.

Furthermore, the versatility of SIMPLE extends beyond viral testing to bacterial sample pooling. The enrichment capability of the SIMPLE effectively reduces Ct values in pooled bacterial samples (*Escherichia coli*, Figure S3, Supporting Information), illustrating its potential for broad expansion and application across various fields (Table S3, Supporting Information). In the context of low‐ and middle‐income countries (LMICs), the SIMPLE technique is particularly promising for enhancing the detection of urinary tract infections (UTIs) and sexually transmitted infections (STIs) through urine sample pooling.^[^ ^21^, ^22^ ^]^ These infections are prevalent in LMICs and necessitate scalable, cost‐effective diagnostic solutions.

SIMPLE's ability to maintain diagnostic accuracy while pooling biological samples can facilitate widespread screening, optimize resource utilization, and improve public health outcomes by enabling early detection and treatment. The integration of the SIMPLE method into diagnostic laboratories and primary care centers can enable optimizing healthcare resources and testing efficiency, providing a robust approach to managing disease infections in large groups or populations. Its broad applicability across different testing scenarios ensures that it will be a vital asset in both routine and emergency testing situations, thereby contributing to improved health and safety outcomes globally.

## 4 Experimental Section

### Red Blood Cell Membrane Isolation

RBCM was isolated from human whole blood anticoagulated with K2EDTA. Initially, plasma and the buffy coat were removed by centrifuging at 800 g for 5 min. The remaining red blood cells (RBCs) were subsequently washed three times with ice‐cold 1X phosphate‐buffered saline (PBS, LB004, DUKSAN, Republic of Korea), with gentle shaking. Subsequently, the washed RBCs were hemolyzed, and the released free hemoglobin was removed by centrifugation at 20 000 g for 30 min. After three additional washing steps, a light pink RBCM pellet was obtained and stored at −80 °C for future use.

### Evaluation of Enrichment Performance of the RBCM‐coated PES Membrane

To evaluate the enrichment performance of the RBCM‐coated PES membrane, the RBCM stock was first adjusted to a 2% (v/v) concentration using distilled water (D.W). This solution was coated onto a 25 mm PES membrane (GVS, 0.03 µm pores), which fits the membrane holder of the stirred cell (341000, STERLITECH, USA). The inactivated SARS‐CoV‐2 (NATtrol SARS‐CoV‐2 Stock External Quality Control, Zeptometrix, Canada) was diluted 300‐fold with 1X PBS, and 3 mL of this diluted sample was injected into the stirred cell for enrichment under 3 bars of pressure for 1 min. Subsequently, to resuspend the enriched virus, 100 µL of 1X PBS was injected into the stirred cell, and the resuspended sample (enriched virus) was collected by pipetting. Viral RNA was subsequently extracted using the TaKaRa MiniBEST Universal RNA Extraction Kit (Takara Bio Inc., Japan) and analyzed via RT‐PCR using the AccuPower RT‐PCR PreMix (BIONEER, Korea).

### Fabrication of SIMPLE

To fabricate the SIMPLE, 40 µL of the 2% RBCM stock solution was applied to a 7 mm PES membrane (punched from a 25 mm PES) and heat‐treated at 50 °C for 30 min on a hot plate. Once the RBCM had completely dried on the PES, this RBCM‐coated PES was placed on top of the silica membrane within the spin column (from the TaKaRa MiniBEST Universal RNA Extraction Kit, Takara Bio Inc., Japan). An O‐ring was subsequently positioned on top of the stacked membranes to secure them together, ensuring no sample leakage occurred through the gap between the column wall and the membrane during the enrichment process.

### Operational Protocol of SIMPLE

To operate the SIMPLE, 500 µL of the pooled sample was first introduced into the SIMPLE column. The sample was enriched by centrifugation at 8 000 rpm, the optimized rpm as detailed in Figure 2. If processing a larger volume of samples was required, this centrifugation process was repeated. After enriching the pooled sample, 200 µL of lysis and binding buffer (provided in the TaKaRa viral RNA extraction column kit) and 200 µL of 1X PBS buffer were added to the spin column. These steps were intended to lyse the enriched virus and extract the released RNA through the silica membrane. All subsequent purification processes were conducted according to the manufacturer's instructions, detailed in Figure S4 (Supporting Information). The final eluted volume of the purified RNA was 30 µL.

### SARS‐CoV‐2 Patient Sample and Sample Pooling

PCR‐confirmed SARS‐CoV‐2 patient samples were acquired from Trina Bioreactives AG (Nänikon, Switzerland), with detailed information available in Table S1 (Supporting Information). For a pool size of 6, one positive sample (200 µL) was mixed with five negative samples (each 200 µL), resulting in a total volume of 1.2 mL. This pooled sample was subsequently enriched and extracted using the SIMPLE column to obtain 30 µL of enriched and purified viral RNA. For larger pooling sizes of 8, 16, 32, 64, and 128, one positive sample (200 µL) was combined with varying volumes of negative viral transport medium (VTM) to achieve the desired dilution levels. Specifically, the total volumes for these pool sizes were 1.6, 3.2, 6.4, 12.8, and 25.6 mL, respectively, corresponding to 8, 16, 32, 64, and 128‐fold dilutions. Each of these pooled samples was processed in 500 µL increments using the SIMPLE column to elute 30 µL of enriched and purified viral RNA. For control purposes, 200 µL of each pooled sample was used.

### Viral RNA Extraction and RT‐PCR

A viral RNA extraction kit (TaKaRa MiniBEST Viral RNA/DNA Extraction Kit, Takara Bio Inc., Japan) was used to obtain purified SARS‐CoV‐2 RNA. RT‐PCR was performed using AccuPower® RT‐PCR PreMix (BIONEER, Korea). In the preparation of the lyophilized PCR mixture, the following components were added: 1 µL of a 5 µm primer set (forward and reverse), 3 µL of viral RNA template, 1 µL of 20X fluorescence dye (Chai Inc., USA), and 15 µL of DW. The primer sequences used were sourced from previous literature (forward primer: 5' TTCGGAAGAGACAGGTACGTTA‐3' and reverse primer: 5'‐AGCAGTACGCACACAATCG‐3'). The thermocycler settings were programmed at 50 °C for 2 min and 95 °C for 10 min, followed by 40 cycles of denaturation at 95 °C for 15 s, and annealing and extension at 60 °C for 1 min.^[^ ^23^ ^]^

### Scanning Electron Microscopy (SEM) Analysis and Imaging

Field emission scanning electron microscopy (FE‐SEM, JSM‐6701F, JEOL, Tokyo, Japan) was employed to characterize the surface of the RBCM‐coated membrane at a magnification of 3300X. Prior to analysis, all membranes were coated with gold to enhance conductivity and image clarity. This coating was performed using an automatic magnetron sputter coater (Au Coater, 108auto, Cressington Scientific Instruments, Watford, United Kingdom) at a current of 10 mA for 120 s.

### Characterization of RBCM‐Coated PES Membrane

XPS analysis was conducted on a Thermo Scientific K‐alpha XPS system (Thermo Scientific Inc., UK) equipped with a Kα micro‐focused monochromator (1486.6 eV) and a variable spot size adjustable from 30 to 400 µm in 5 µm increments, to assess elemental composition changes on the surface of the RBCM‐coated PES membrane. The system achieved an energy resolution of FWHM ≤ 0.5 eV for Ag 3d₅/₂, measured with a 180° double‐focusing hemispherical analyzer and a 128‐channel detector. The sample chamber was maintained at a base pressure of 4.8×10⁻⁹ mbar, with charge compensation provided by a dual‐beam source. An ion gun with an energy range of 100–4000 eV was used for surface charge neutralization, facilitating the analysis of insulating materials. The instrument was configured for angle‐resolved measurements, varying angles from 0° to 70°. To assess the potential non‐specific binding of RNA to the RBCM‐coated PES membrane, 500 µL of RNA (10 ng µL^−1^) was added to a column equipped with RBCM‐coated PES membrane. Following a 5‐minute incubation, the column was centrifuged at 8 000 RPM, and the RNA concentration in the eluate was measured using a NanoDrop spectrophotometer (NanoDrop 2000, ThermoFisher Scientific Inc., USA).

### Bacterial DNA Extraction and PCR

*E. coli* culture (K‐12 Strain, wild type) was acquired from Biozoa Biological Supply Co., Korea. Bacterial DNA was extracted using the TaKaRa MiniBEST Bacteria Genomic DNA Extraction Kit Ver.3.0 (Takara Bio Inc., Japan). PCR analysis of bacterial DNA was conducted using TB Green Premix Ex Taq™ II (Tli RNaseH Plus) (Takara Bio Inc., Japan). This included DNA obtained from the heat‐treated bacterial lysate, purified bacterial DNA from the original column, and enriched/purified bacterial DNA from the SIMPLE column. The PCR reagent mix was prepared with 10 µL of 2X TB Green Premix Ex Taq II I (Tli RNaseH Plus), 1 µL of a 5 µm primer set (forward and reverse), 1.5 µL of bacterial lysate or DNA template, and 7.5 µL of D.W. Primer sequences were sourced from previous literature (forward primer: 5’‐TTGAAAATGGTCTGCTGCT‐3’ and reverse primer: 5’‐TATTGGCTTCATCCACCACA‐3’). The thermocycler conditions were set to 95 °C for 3 min, followed by 40 cycles of denaturation at 95 °C for 30 s, annealing at 58 °C for 30 s, extension at 72 °C for 30 s, and a final extension at 72 °C for 10 min.^[^ ^24^ ^]^

## Conflict of Interest

The authors declare no conflict of interest.

## Supporting information



Supporting Information

## Data Availability

The data that support the findings of this study are available from the corresponding author upon reasonable request.
