# Supplementary material for: Enhancing RT‐PCR Throughput and Sensitivity through Large‐Scale Sample Pooling Using a Nano‐Hybrid Membrane
Source: Adv Sci (Weinh). 2025 Jan 20;12(10):2408771. doi: 10.1002/advs.202408771 (PMC11904979; doi:10.1002/advs.202408771)
Supplement: Supplementary file 1 — Supporting Information [file ADVS-12-2408771-s001.docx]

Supporting Information

Enhancing RT-PCR Throughput and Sensitivity Through Large-Scale Sample Pooling Using a Nano-Hybrid Membrane

*Na Eun Lee, Kang Hyeon Kim, Ji Hye Hong, Seungmin Lee, Jeong Soo Park, Dohwan Lee*, Dae Sung Yoon* and Jeong Hoon Lee**

**The PDF file includes:**

Figure S1 to S4

Table S1 to S3

Supporting Figures


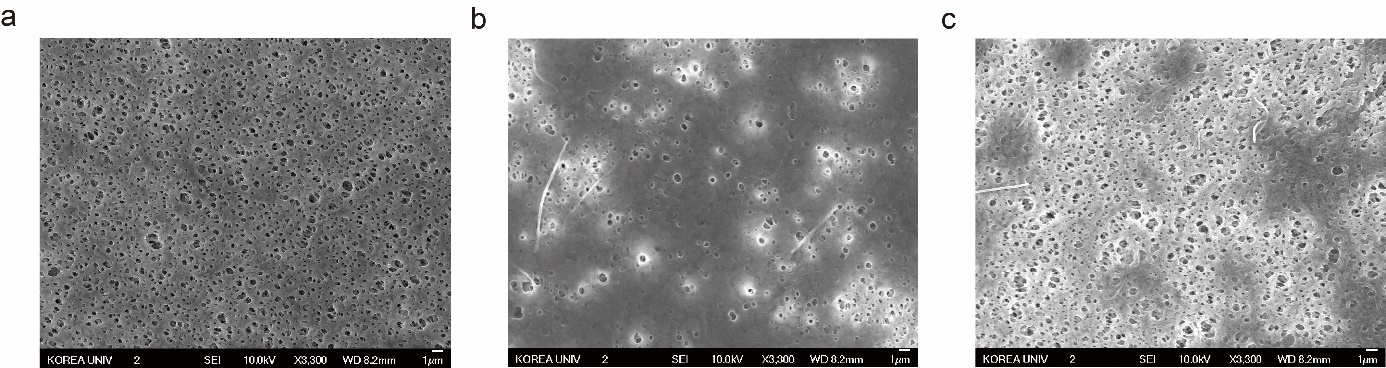


Figure S1. Scanning electron microscopy images. Surface of a) PES membrane (average pore size = 30 nm), b) RBCM-coated PES membrane (30 nm), and c) RBCM-coated PES membrane after lysis buffer treatment.


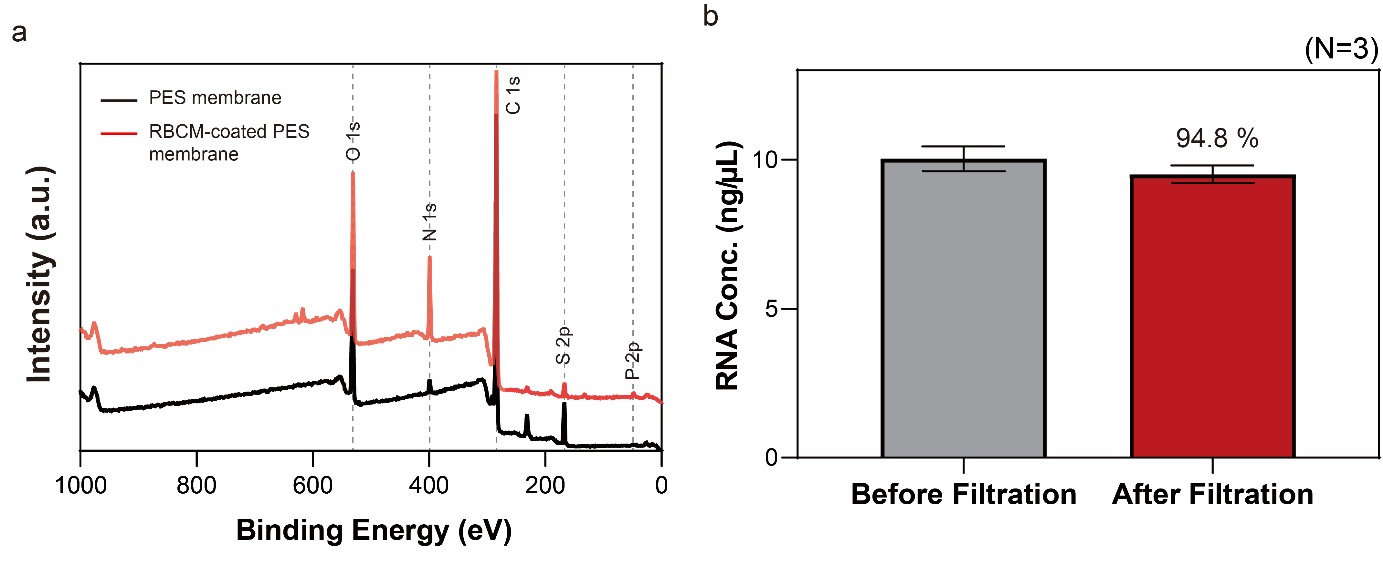


Figure S2. Characterization of the RBCM-coated PES membrane. a) X-ray Photoelectron Spectroscopy (XPS) spectra comparing the surface composition of the uncoated (black line) versus the RBCM-coated PES membrane (red line). The additional peaks for phosphorus and nitrogen observed in the RBCM-coated membrane confirm the presence of biomolecular elements introduced by RBCM coating. (b) RNA concentration measured after filtration through the RBCM-coated PES membrane, demonstrating negligible non-specific binding at ~ 5%.


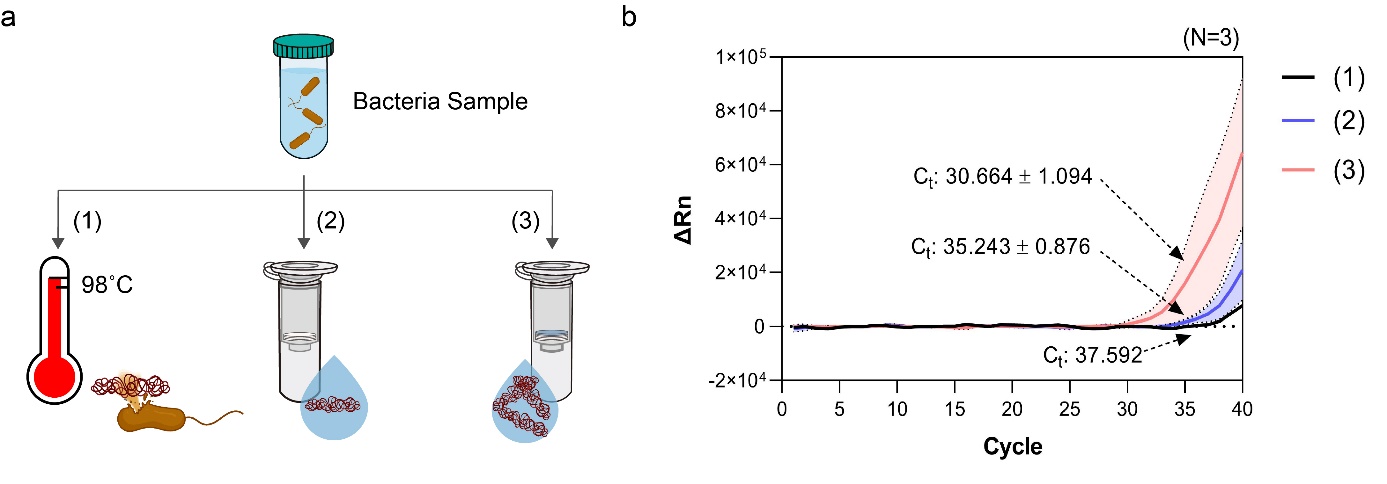


Figure S3. Application of SIMPLE to bacterial sample. a) *Escherichia coli* (*E. coli*) sample was (1) heat-lysed at 98 °C for 10 min, (2) bacterial DNAs were extracted using a conventional column, and (3) *E. coli* was enriched and extracted using the SIMPLE column. b) Ct values obtained by RT-PCR for these samples. The errors (red and blue spread) represent the standard deviation (N = 3).


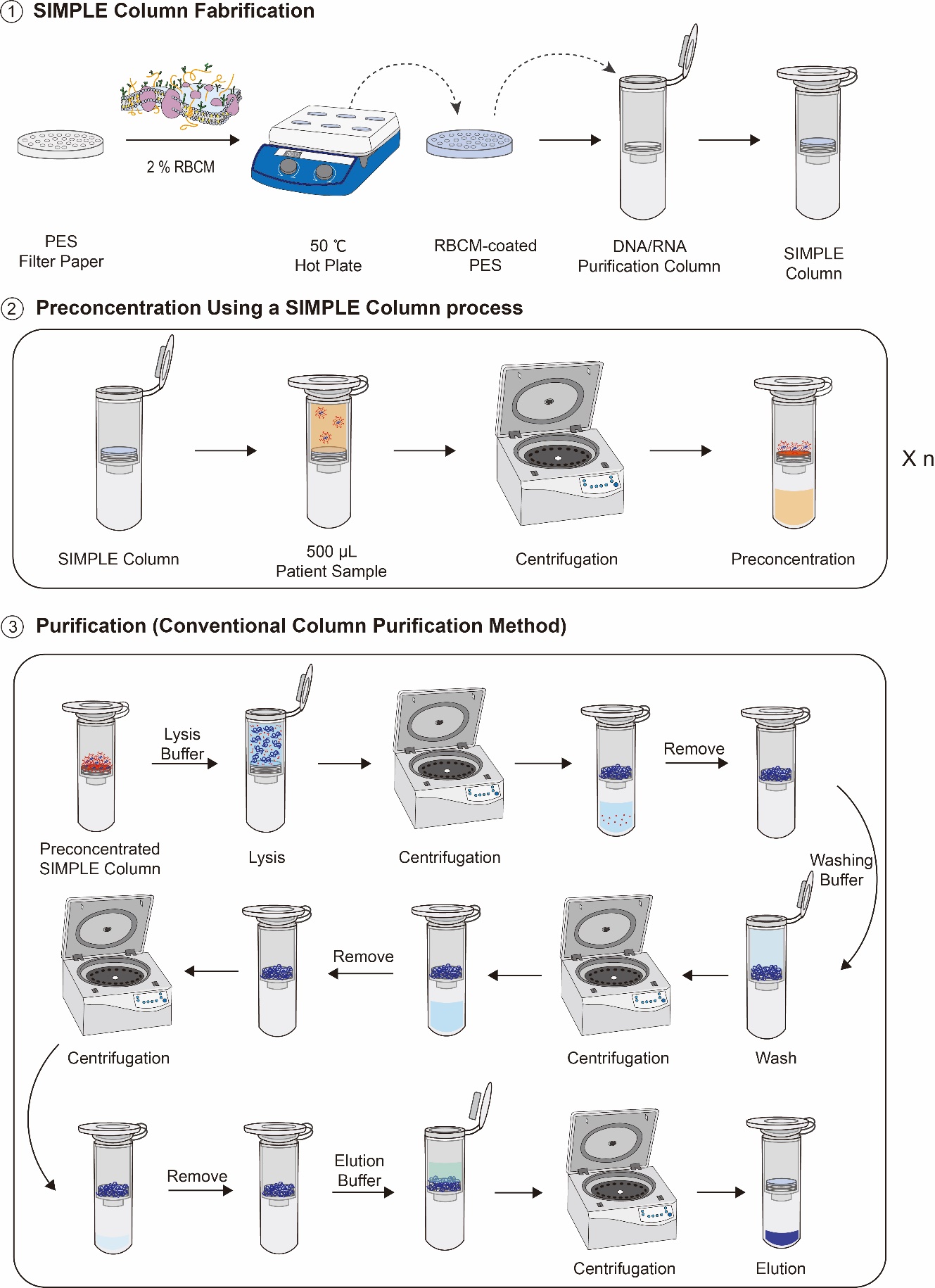


Figure S4. Schematic of the process of the SIMPLE column. Fabrication, preconcentration, and RNA extraction of the SIMPLE column.

Supporting Table

Table S1. Information on COVID-19 positive patient samples.

| Sample No. | Age | Gender | Symptom  Onset Date | Date of Collection | Collection method | PCR test device | Anatomical collection site 1 | Anatomical collection site 2 |
| --- | --- | --- | --- | --- | --- | --- | --- | --- |
| P1 | 55 | F | 08.Nov.20 | 09.Nov.20 | SWABX2 | BioRad CFX 384  Thermocycler | Nasopharynx | Nares Collection Site |
| P2 | 31 | F | 30.Mar.21 | 31.Mar.21 | SWABX2 | BioRad CFX 384  Thermocycler | Nasopharynx | Nares Collection Site |
| P3 | 34 | M | 12.Apr.21 | 12.Apr.21 | SWABX2 | BioRad CFX 384  Thermocycler | Nasopharynx | Nares Collection Site |
| P4 | 58 | M | 14.Apr.21 | 14.Apr.21 | SWABX2 | BioRad CFX 384  Thermocycler | Nasopharynx | Nares Collection Site |
| P5 | 51 | M | 13.Apr.21 | 13.Apr.21 | SWABX2 | BioRad CFX 384  Thermocycler | Nasopharynx | Nares Collection Site |
| P6 | 19 | F | 28.Feb.21 | 01.Mar.21 | SWABX2 | BioRad CFX 384  Thermocycler | Nasopharynx | Nares Collection Site |
| P7 | 18 | M | 27.Feb.21 | 01.Mar.21 | SWABX2 | BioRad CFX 384  Thermocycler | Nasopharynx | Nares Collection Site |
| P8 | 16 | M | 11.Apr.21 | 12.Apr.21 | SWABX2 | BioRad CFX 384  Thermocycler | Nasopharynx | Nares Collection Site |

Table S2. Comparison of SIMPLE with other commercial purification kits

| Product Name | Company | Method | Sample  Volume | Processing  Time | Target  Preconcentration | Final  Extraction Volume |
| --- | --- | --- | --- | --- | --- | --- |
| DiagSpin Virus DNA/RNA Silica Spin Column | CD Bioparticles | Centrifugation | 200 µL | 30 min | X | 20-150 µL |
| DiagSpin Bacteria DNA Silica  Spin Column, Rapid | CD Bioparticles | Centrifugation | 0.5-2 mL | 40 min | X | 100 µL |
| MagIso DNA&RNA Magnetic  Silica Particles | CD Bioparticles | Magnetic Particles | 200 µL | ~ 30 mins | X | ~ 20-100 µL |
| TaKaRa MiniBEST Viral  RNA/DNA Extraction Kit | TaKaRa | Centrifugation | 200 µL | 20 min | X | 30-50 µL |
| MagListo™ 5M Genomic DNA Extraction Kit | BIONEER | Magnetic Particles | 200 µL | 20 min | X | 100 µL |
| SIMPLE (This work) | This Work | Centrifugation | 200 µL -25.6 mL | 20-45 min | O | 30 µL |

Table S3. Potential applications of SIMPLE in diagnosing various targets

| Target | Disease | Sample Type | Pooling | ref. |
| --- | --- | --- | --- | --- |
| SARS-CoV-2 | COVID-19 | VTM | Required | [1] |
| *Escherichia coli* | Urinary tract infection | Urine/ UTM | Required | [2] |
| Influenza A, B virus | Flu | VTM | Required | [3] |
| Human Immunodeficiency Virus-1, 2 | Acquired Immune Deficiency Syndrome | Saliva | Required | [4] |
| *Chlamydia trachomatis* | Sexually transmitted infection | Urine/ UTM | Required | [5] |

References

[1] L. Mutesa, P. Ndishimye, Y. Butera, J. Souopgui, A. Uwineza, R. Rutayisire, E. L. Ndoricimpaye, E. Musoni, N. Rujeni, T. Nyatanyi, E. Ntagwabira, M. Semakula, C. Musanabaganwa, D. Nyamwasa, M. Ndashimye, E. Ujeneza, I. E. Mwikarago, C. M. Muvunyi, J. B. Mazarati, S. Nsanzimana, N. Turok, W. Ndifon, *Nature* **2021**, *589* (7841), 276, <https://doi.org/10.1038/s41586-020-2885-5>.

[2] A. Snyder Jennifer, J. Haugen Brian, L. Buckles Eric, C. V. Lockatell, E. Johnson David, S. Donnenberg Michael, A. Welch Rodney, L. T. Mobley Harry, *Infection and Immunity* **2004**, *72* (11), 6373, <https://doi.org/10.1128/iai.72.11.6373-6381.2004>.

[3] T. Van Tam, J. Miller, M. Warshauer David, E. Reisdorf, D. Jernigan, R. Humes, A. Shult Peter, *Journal of Clinical Microbiology* **2020**, *50* (3), 891, <https://doi.org/10.1128/jcm.05631-11>.

[4] C. van Schalkwyk, J. Maritz, G. U. van Zyl, W. Preiser, A. Welte, *BMC Infectious Diseases* 2019, *19* (1), 136, <https://doi.org/10.1186/s12879-019-3767-z>.

[5] Y. Xu, L. Aboud, E. P. F. Chow, M. B. Mello, T. Wi, R. Baggaley, C. K. Fairley, R. Peeling, J. J. Ong, International Journal of Infectious Diseases 2022, 118, 183, https://doi.org/https://doi.org/10.1016/j.ijid.2022.03.009.
